# Hierarchical Surrogate Modeling and Optimization of the SO_2_ Recovery–Water Loss Trade-Off in Basic Aluminum Sulfate Regeneration

**DOI:** 10.3390/molecules31193468

**Published:** 2026-09-29

**Authors:** Zijun Wang, Bo Wang, Jianmin Li, Junming Wen, Ping Wang, Gao Wen, Xiangming Xu, Wenlin Qiu, Zhoufeng Bian, Zhenkun Sun, Dennis Lu

**Affiliations:** 1 Key Laboratory of Energy Thermal Conversion and Control, Ministry of Education, School of Energy and Environment, Southeast University, Nanjing 210096, China; m13884347632@163.com (Z.W.);; 2 Inner Mongolia Juda Power Generation Co., Ltd., Ordos 014300, China; 3 Inner Mongolia Jince Ecological Technology Co., Ltd., Hohhot 010000, China; 4 School of Energy and Power Engineering, Xi’an Jiaotong University, Xi’an 710049, China

**Keywords:** basic aluminum sulfate, vacuum regeneration, SO_2_ recovery, water loss, multiobjective optimization

## Abstract

Vacuum regeneration can close the absorbent loop in basic aluminum sulfate (BAS) wet flue-gas desulfurization and SO_2_ capture, but SO_2_ release occurs together with energy-intensive water evaporation. This study developed a response-specific framework to predict and balance these coupled rates using existing vacuum-regeneration data. The SO_2_ model included absolute pressure, temperature, aluminum concentration, basicity, and sulfite concentration, whereas the H_2_O model used pressure and temperature. Candidate regressors were assessed by cross-validation, error statistics, and physical-trend consistency. Gaussian process regression with a Matérn-5/2 automatic-relevance-determination kernel best predicted SO_2_ desorption (RMSE = 0.0631; R^2^ = 0.9746), while an automatic-relevance-determination squared-exponential kernel best predicted H_2_O evaporation (RMSE = 0.0793; R^2^ = 0.9928). Sulfite concentration dominated the SO_2_ response; pressure and temperature controlled both rates. Lower pressure and higher temperature improved SO_2_ recovery but increased water loss. Pareto analysis identified a model-supported compromise at approximately 26–30 kPa and 340–342 K. A 120 min recursive simulation showed declining instantaneous SO_2_ release as sulfite was depleted, while water evaporation continued. Within the experimental domain, the framework supports joint selection of pressure, temperature, and operating time for BAS regeneration.

## 1. Introduction

Wet flue-gas desulfurization can remove SO_2_ efficiently from large combustion-gas streams, yet high removal efficiency does not by itself ensure a sustainable process. Conventional systems may transfer the environmental burden from the stack to continued mineral and water consumption, auxiliary-energy demand, wastewater, and solid by-products. Limestone–gypsum scrubbing remains the industrial reference because it is robust and well established, but limestone preparation, forced oxidation, gypsum separation, and wastewater treatment add resource and energy requirements; moreover, gypsum has value only where its quality and local market permit beneficial use [1,2]. Dry and semidry routes reduce liquid effluent, although their performance and water demand differ from those of wet systems [3,4]. Regenerative absorption offers another path: a concentrated SO_2_ stream is recovered and the lean absorbent is returned to the absorber [5,6]. The practical question is therefore not simply whether SO_2_ can be captured, but whether the absorbent can be regenerated without excessive solvent loss or energy consumption.

This question is especially relevant to vacuum regeneration of SO_2_-rich basic aluminum sulfate (BAS) solution. A stronger vacuum and a higher temperature favor SO_2_ release, but the same changes accelerate water evaporation. The vapor load increases condensation duty, reduces the liquid inventory, and alters the apparent concentrations of nonvolatile solutes. An operating point selected solely for a high instantaneous SO_2_ rate may consequently impose an unfavorable water and energy penalty, and its advantage may diminish as the solution composition changes. The present study therefore asks how the two responses can be predicted and jointly optimized when their experimental datasets have unequal dimensions.

Regenerative SO_2_ capture has been investigated with sodium sulfite, organic amines, magnesium-based media, ionic liquids, and deep eutectic solvents [6,7,8,9,10]. Collectively, this literature demonstrates that absorbent reuse and concentrated sulfur recovery are feasible, while also showing why capture capacity cannot be the only selection criterion. Sulfite solutions may oxidize or crystallize; amine systems can incur sensible- and latent-heat duties as well as volatility and degradation losses; some magnesium processes require relatively high regeneration temperatures; and the viscosity, cost, and scale-up of ionic liquids and deep eutectic solvents remain important constraints [6,7,8,9,10]. These findings place regeneration intensity, chemical stability, solvent retention, and downstream recovery on the same footing as absorption performance.

BAS is a regenerative medium that may provide a useful balance among these requirements. In its mildly acidic aluminum-containing solution, absorbed SO_2_ undergoes dissolution, hydration, acid–base dissociation, and reversible association with active aluminum species, producing an S(IV)-rich liquid. Heating or lowering the total pressure shifts the coupled equilibria toward molecular SO_2_, allowing the absorbent to be recycled [11,12,13]. Previous studies have established the feasibility of BAS absorption and described the effects of temperature, aluminum concentration, basicity, gas–liquid contact, and inlet SO_2_ concentration [11,12,13]. Related kinetic work has shown that S(IV) oxidation depends on temperature, oxygen partial pressure, aluminum concentration, and basicity, and that ethylene glycol can inhibit this side reaction [14,15],. This evidence provides the chemical basis for a cyclic BAS process.

The regeneration step has also received substantial attention. Vacuum desorption can release SO_2_ at lower temperatures and has been used to examine the effects of pressure, temperature, agitation, and solution composition [16]. Falling-film equipment improves interfacial renewal and heat and mass transfer, with converging–diverging and spring-tube designs providing further intensification [17,18,19]. Microwave-assisted regeneration has been explored as another means of accelerating dissociation and SO_2_ release [20]. These studies identify the variables and equipment features that control regeneration, but most evaluate a specific device or a single SO_2_-centered performance measure under fixed conditions.

A central gap is the limited treatment of water evaporation as a competing response. The low-pressure, high-temperature region that promotes SO_2_ release also produces a substantial water-vapor load. In BAS vacuum regeneration, water vaporization has been reported to contribute more than 85% of total regeneration energy use [16]. Beyond its latent-heat requirement, evaporation increases condenser duty and changes liquid volume and composition throughout a run. Thus, an optimum based only on SO_2_ desorption efficiency or an instantaneous SO_2_ surface may be energetically misleading. The literature has not yet resolved this SO_2_–H_2_O compromise through multiobjective analysis and then carried it into a time-dependent material balance for BAS regeneration.

A second gap arises from the available data. A mechanistic description would require internally consistent thermophysical properties, vapor–liquid equilibria, S(IV) speciation, reversible-reaction parameters, and gas–liquid transport coefficients over the full operating range. Those inputs are not sufficiently complete. The experimental record is also unbalanced: SO_2_-rate measurements include pressure, temperature, aluminum concentration, basicity, and sulfite concentration, whereas H_2_O-rate measurements primarily include pressure and temperature. Forcing both responses into one input space would either invent unsupported composition effects for water or discard valid composition information for SO_2_. Either choice would weaken the analysis.

Surrogate modeling offers a defensible middle ground between isolated single-factor correlations and an underdetermined first-principles model. Within a sampled domain, a surrogate can reproduce nonlinear behavior, rank predictors, and support rapid optimization [21,22]. Gaussian process regression (GPR) is particularly useful for small process datasets because it represents smooth nonlinear responses and supplies a probabilistic prediction structure [22,23]. High fit statistics alone are insufficient, however: sparse sampling and kernel choice can produce unstable or physically implausible surfaces. Model selection should therefore combine cross-validation with checks of the expected responses to pressure, temperature, and composition. Because the two outputs are supported by different evidence, separate response-specific models are more credible than a uniform model with a common input vector.

Accordingly, this work develops a hierarchical framework for predicting and balancing SO_2_ desorption and H_2_O evaporation during BAS vacuum regeneration. Linear, polynomial, support-vector, tree-based, ensemble, and Gaussian-process regressors are screened independently for each response using only experimentally supported predictors. The selected models are then used to identify influential variables, map the shared pressure–temperature domain, and define a Pareto region that improves SO_2_ release without ignoring water loss. Recursive material balances connect the instantaneous rate predictions to cumulative SO_2_ recovery, water evaporation, liquid volume, and composition. The analysis is intentionally restricted to interpolation and process screening within the experimental domain; it neither replaces equilibrium and transport modeling nor supports extrapolation to untested equipment or solution chemistry.

## 2. Results and Discussion

### 2.1. Experimental-Data-Based Results and Model Reliability

#### 2.1.1. SO_2_-Desorption-Rate Surrogate

**Figure 1:**
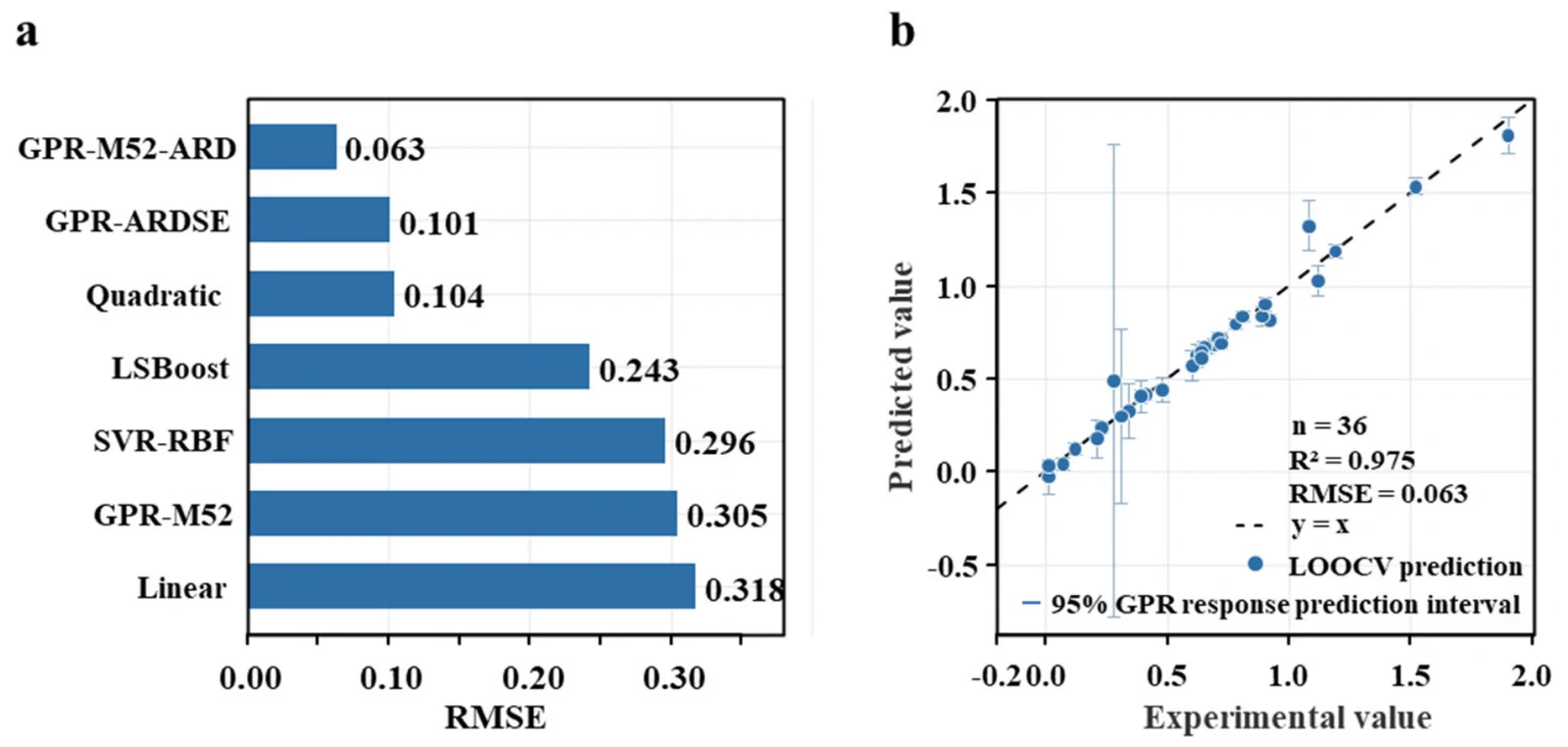
(**a**) Comparison of candidate models for predicting the SO_2_ desorption rate; (**b**) agreement between experimental and GPR-M52-ARD-predicted SO_2_ desorption rates.

Candidate-model errors for the SO_2_ rate are compared in Figure 1a. Every model captured some input–output structure, but the error levels varied markedly. GPR-M52-ARD gave the smallest RMSE among the tested methods, whereas the linear, polynomial, SVR, tree, and ensemble models produced larger errors or less stable response surfaces. The result indicates that the SO_2_ data contain smooth nonlinear relationships and that allowing separate length scales for individual predictors improved the fit.

Figure 1b shows that GPR-M52-ARD predictions cluster around the 1:1 line. The selected SO_2_ model achieved an LOOCV RMSE of 0.0631 and R^2^ = 0.9746, with RMSE expressed in units of 10^−5^ mol·s^−1^. The figure additionally shows the pointwise 95% GPR response prediction intervals associated with the leave-one-out predictions. The interval widths vary among observations because predictive uncertainty depends on the local information available to the fitted GPR model. Residuals increased in the high-rate region, where the original experiments were sparse, so predictions near that boundary warrant more caution than those in densely sampled regions.

GPR’s performance is consistent with its ability to model smooth nonlinear behavior and quantify predictive uncertainty in small datasets [22,23],. Even so, the model should be understood as an interpolator rather than as evidence of a fully identified mechanism. Its value here is accurate local prediction and a physically credible response surface within the measured domain.

#### 2.1.2. H_2_O-Evaporation-Rate Surrogate

**Figure 2:**
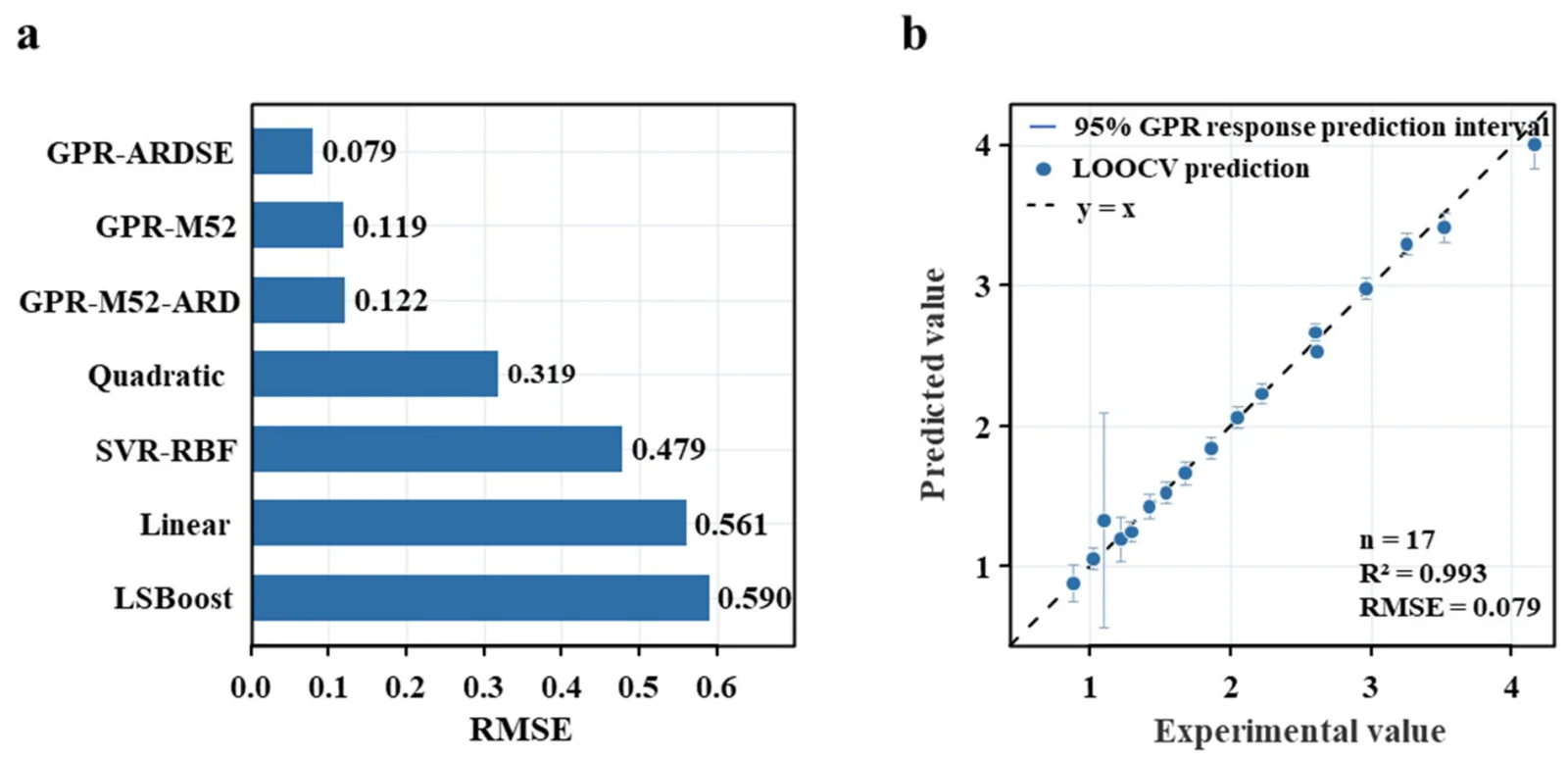
(**a**) Comparison of candidate models for predicting the H_2_O evaporation rate; (**b**) agreement between experimental and GPR-ARDSE-predicted H_2_O evaporation rates.

Figure 2a presents the H_2_O model comparison. GPR-ARDSE achieved the lowest RMSE (0.079), compared with 0.119 for GPR-M52 and 0.122 for GPR-M52-ARD; the linear, polynomial, SVR, and tree-based alternatives were less accurate or less stable. The smoother squared-exponential structure was therefore better suited to the observed pressure–temperature response than the alternatives tested.

Measured and predicted H_2_O rates lie close to the 1:1 line in Figure 2b. The selected H_2_O model achieved an LOOCV RMSE of 0.0793 and R^2^ = 0.9928, with RMSE expressed in units of 10^−4^ mol·s^−1^. Pointwise 95% GPR response prediction intervals are shown with the corresponding leave-one-out predictions in the figure. As for the SO_2_ model, the interval width is input-dependent and reflects the predictive distribution of the fitted GPR model at each held-out condition. These intervals should therefore be interpreted as model-conditioned response prediction intervals rather than confidence intervals for the mean response. Because the dataset is small, the leave-one-out result is more informative than the training fit alone, although it remains an internal validation measure.

Only *P* and *T* were included in the H_2_O model because those were the available predictors. Its high predictive accuracy does not prove that aluminum concentration, basicity, or sulfite concentration has no effect on evaporation; rather, any such dependence remains uncertain and requires dedicated measurements.

### 2.2. Interpretation of Coupled Regeneration Behavior

#### 2.2.1. Effects of Pressure and Temperature

**Figure 3:**
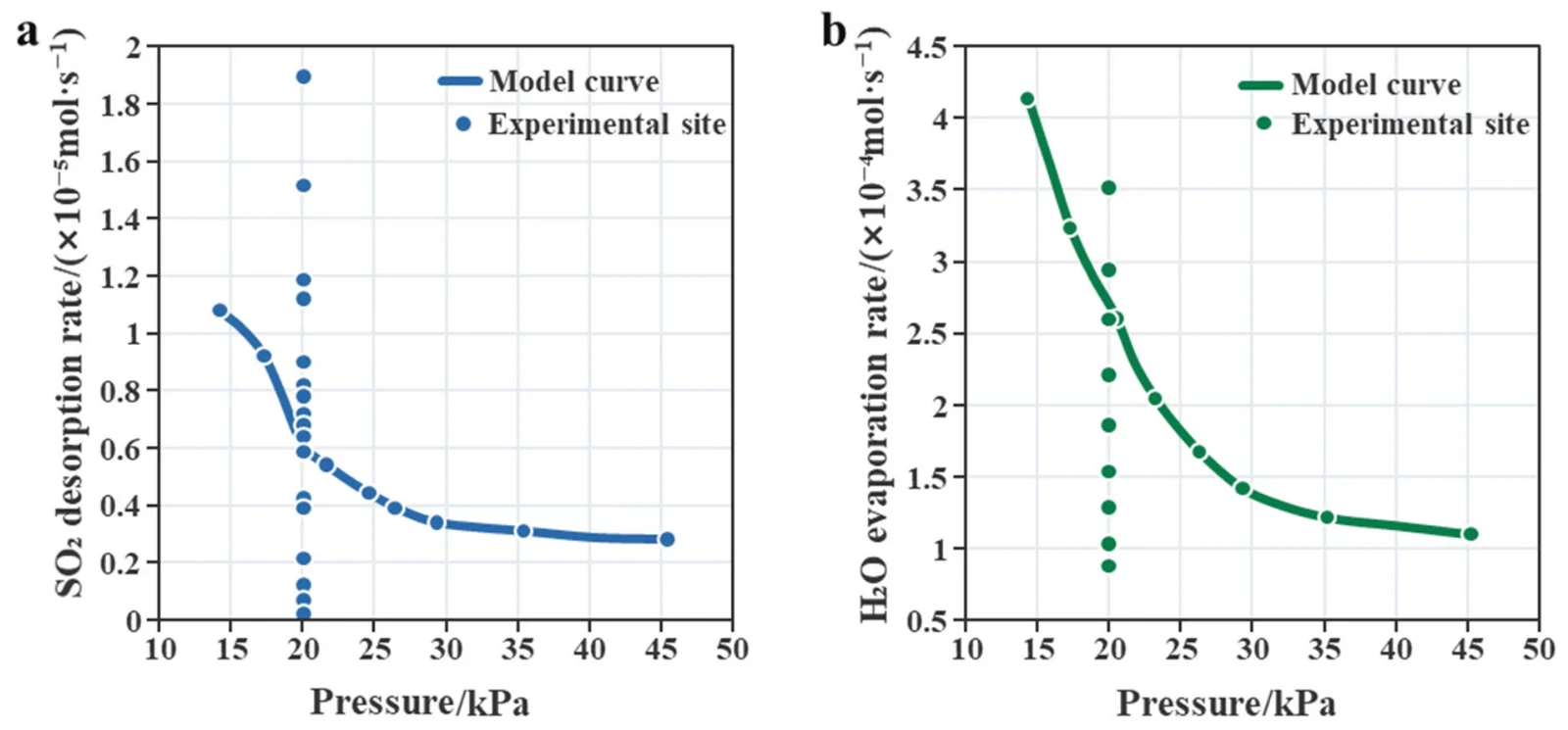
Pressure–response curves for (**a**) SO_2_ desorption and (**b**) H_2_O evaporation. Predictions were evaluated at *T* = 340 K, *c*Al = 15 g·L^−1^, *B* = 25%, and *c*SO_3_^2−^ = 0.06 mol·L^−1^. Experimental points from the corresponding pressure series are shown for direct comparison; observations obtained under other fixed conditions are not treated as validation of this response slice.

**Figure 4:**
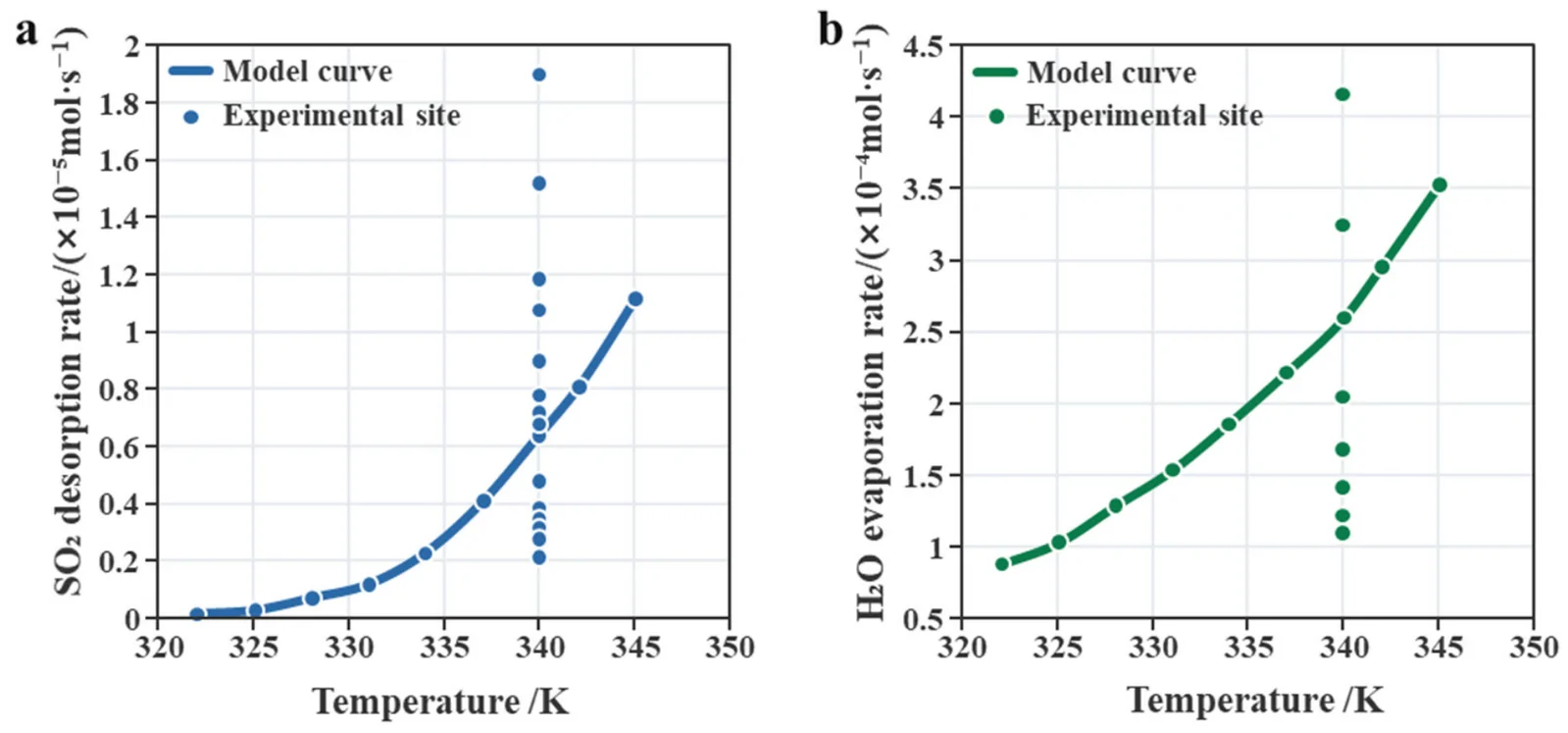
Temperature–response curves for (**a**) SO_2_ desorption and (**b**) H_2_O evaporation. Predictions were evaluated at *P* = 20 kPa, *c*Al = 15 g·L^−1^, *B* = 25%, and *c*SO_3_^2−^ = 0.06 mol·L^−1^. Experimental points from the corresponding temperature series are shown for direct comparison; observations obtained under other fixed conditions are not treated as validation of this response slice.

Figure 3 and Figure 4 compare predicted pressure and temperature responses with the experimental distributions at the baseline composition. Reducing pressure from 45 kPa to about 15 kPa produced the largest increases in both SO_2_ desorption and H_2_O evaporation. Additional vacuum below roughly 15 kPa yielded progressively smaller gains. This diminishing response is operationally important: deeper vacuum continues to consume equipment capacity and promote evaporation even after the incremental SO_2_ benefit has begun to fall.

Raising temperature increased both rates. The SO_2_ response became steeper in the upper temperature range, consistent with enhanced gas transfer and a shift in dissolved S(IV) toward releasable molecular SO_2_. Water evaporation rose concurrently because the vapor-pressure driving force increased. Heating therefore improves SO_2_ recovery while simultaneously increasing water evaporation, with corresponding qualitative implications for regeneration energy demand.

The directions of these responses agree with earlier BAS vacuum and falling-film studies [16,17,18,19]. The present analysis adds two qualifications. Pressure reduction has a region of diminishing marginal benefit, and temperature cannot be evaluated from SO_2_ behavior alone because H_2_O follows the same favorable direction. These coupled observations explain why the strongest vacuum and highest temperature are not automatically the best operating choices.

#### 2.2.2. Effects of Solution Composition

**Figure 5:**
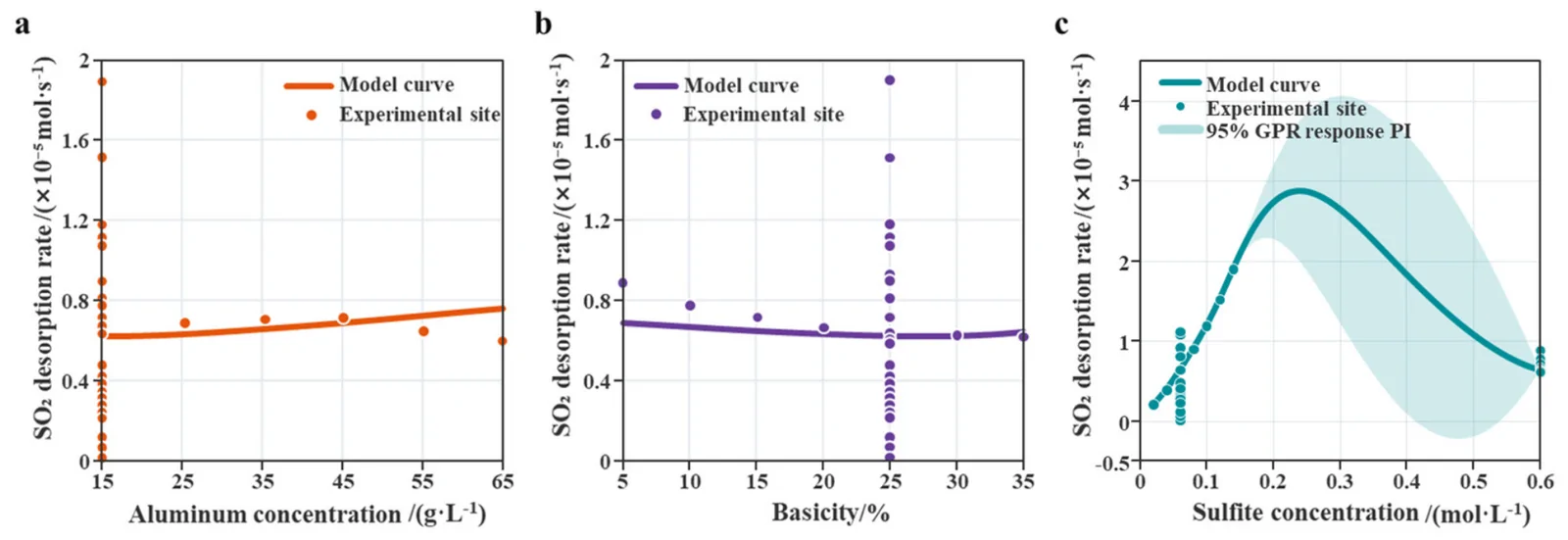
SO_2_-desorption responses to solution composition under source-matched conditions: (**a**) aluminum concentration, evaluated at *P* = 20 kPa, *T* = 340 K, *B* = 25%, and *c*SO_3_^2−^ = 0.6 mol·L^−1^; (**b**) basicity, evaluated at *P* = 20 kPa, *T* = 340 K, *c*Al = 15 g·L^−1^, and *c*SO_3_^2−^ = 0.6 mol·L^−1^; and (**c**) sulfite concentration, evaluated at *P* = 20 kPa, *T* = 340 K, *B* = 25%, and *c*Al = 15 g·L^−1^. Only observations collected under the corresponding held-fixed conditions are used for direct comparison with each response slice.

The SO_2_ composition responses are summarized in Figure 5. Across 15–65 g·L^−1^, increasing aluminum concentration caused only a small predicted change, with a weak upward tendency. Aluminum speciation influences the binding environment of dissolved S(IV), but within this sampled range it was not the main control on the instantaneous desorption rate.

Increasing basicity produced a slight decrease in *r*SO_2_ before the response approached a plateau. Greater basicity can stabilize dissolved S(IV) and reduce the fraction readily released as molecular SO_2_. The modest magnitude of this response indicates that pressure, temperature, and sulfite inventory exerted stronger control under the regeneration conditions studied.

Sulfite concentration had the largest composition effect. At low *c*SO_3_^2−^, the predicted SO_2_ rate rose rapidly as more releasable sulfur became available; after further concentration increase, the response declined. The downturn may reflect changes in speciation, nonideal solution behavior, or transfer resistance, but the present data cannot distinguish these mechanisms. It should therefore be treated as an empirical feature of the fitted domain rather than a universal mechanistic law. In particular, no sulfite concentration was sampled between 0.14 and 0.60 mol·L^−1^. The apparent maximum within this interval is therefore model-derived and should be interpreted together with the pointwise prediction interval rather than as an experimentally confirmed optimum.

The weak aluminum response may seem inconsistent with absorption studies in which aluminum speciation and basicity strongly affected SO_2_ capture [11,12,13]. The two findings address different stages. During absorption, composition controls chemical capacity and uptake; during regeneration, pressure, temperature, and the existing sulfite inventory can dominate the rate at which stored S(IV) is released. This comparison shows why absorber variables cannot be assumed to have the same ranking in the regenerator.

#### 2.2.3. Variable Importance and Pressure–Temperature Response Surfaces

**Figure 6:**
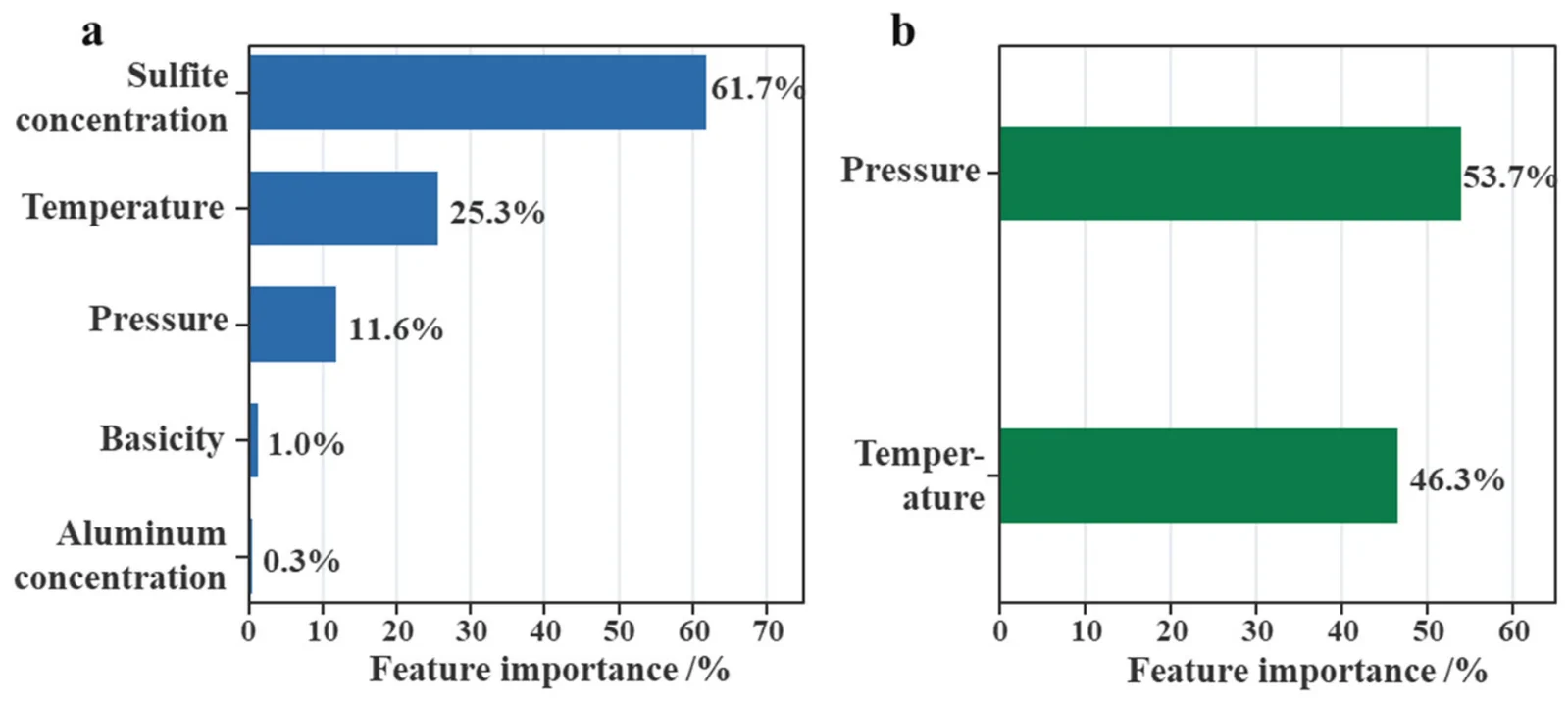
Feature-importance analysis for (**a**) the SO_2_-desorption-rate model and (**b**) the H_2_O-evaporation-rate model.

Permutation feature-importance analysis (Figure 6) yielded relative importance values of 61.7% for sulfite concentration, 25.3% for temperature, 11.6% for pressure, 1.0% for basicity, and 0.3% for aluminum concentration in the SO_2_ model. For the H_2_O model, pressure and temperature accounted for 53.7% and 46.3%, respectively. These values were normalized separately within each response model and therefore describe within-model predictive associations. Because the two models use different datasets and predictor sets, the percentages should not be directly compared in magnitude across the SO_2_ and H_2_O responses. They are permutation-based diagnostics rather than ARD length-scale scores or causal coefficients.

**Figure 7:**
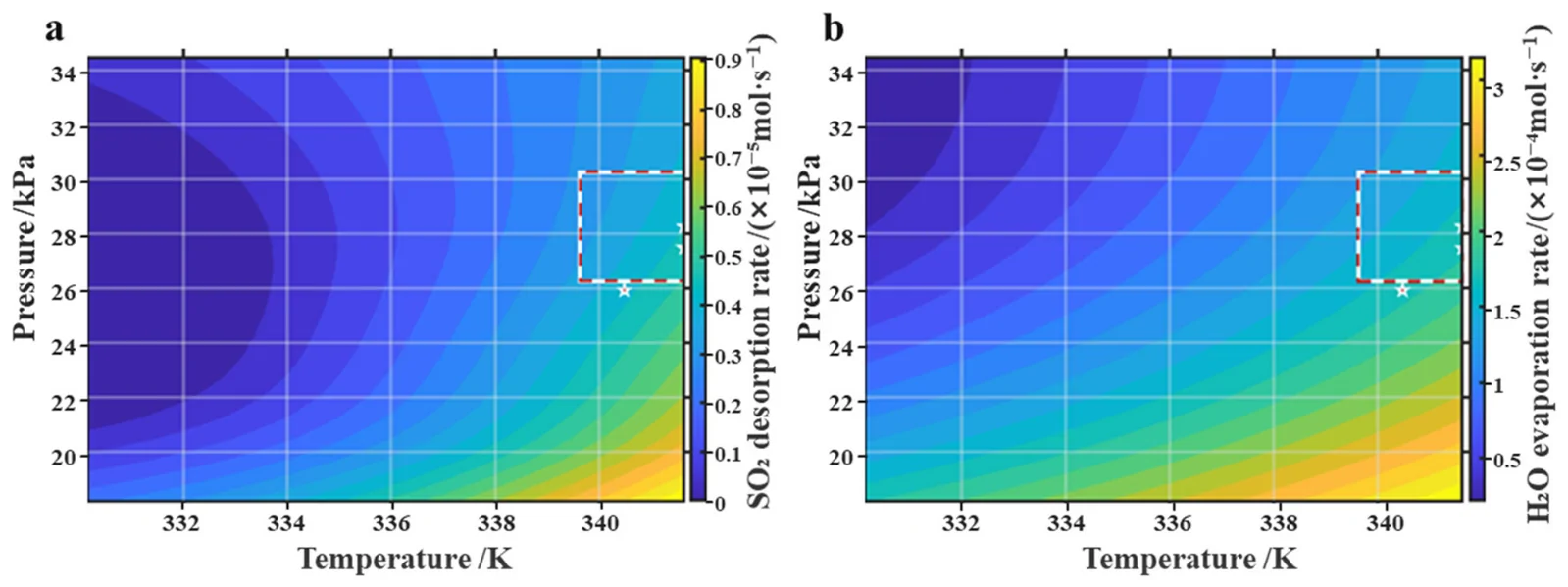
Pressure–temperature distributions of (**a**) the SO_2_ desorption rate and (**b**) the H_2_O evaporation rate.

The response surfaces in Figure 7 make the conflict explicit. High SO_2_ rates and high H_2_O rates occupy substantially overlapping regions as temperature rises and pressure falls. The overlap is a direct reason to replace single-objective SO_2_ maximization with a compromise analysis: every move toward stronger instantaneous regeneration must be evaluated against the accompanying vapor load.

#### 2.2.4. Pareto Trade-Off and Recommended Operating Window

**Figure 8:**
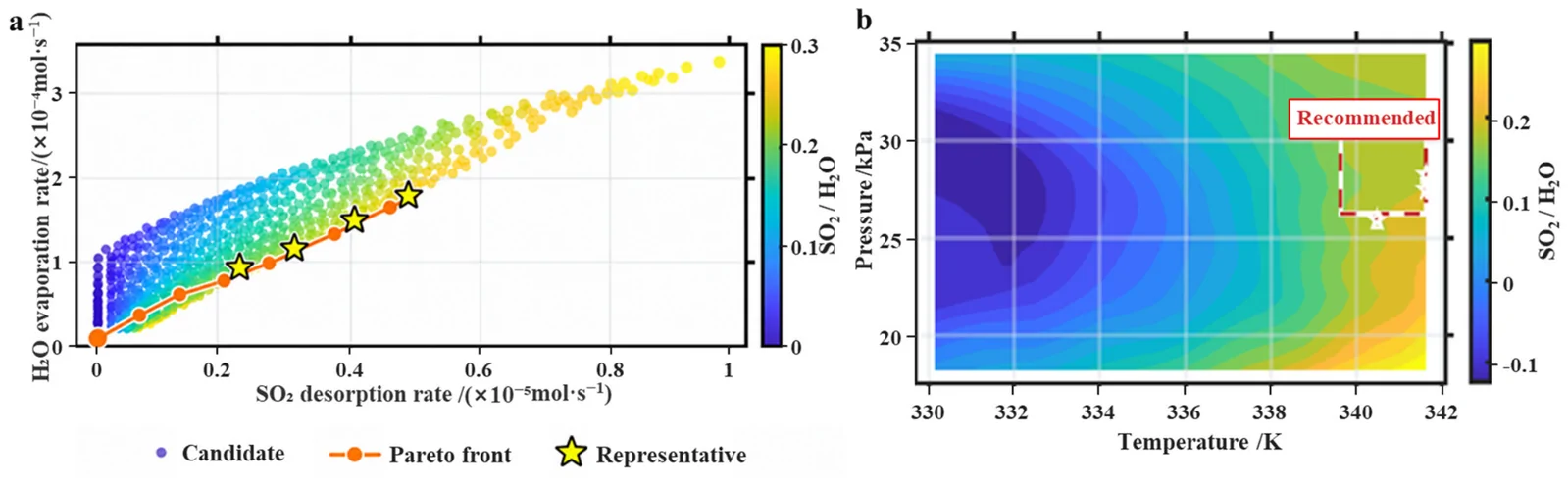
(**a**) Pareto front for the SO_2_-desorption/H_2_O-evaporation trade-off; (**b**) distribution of the recommended operating window.

The Pareto front in Figure 8a confirms that no tested operating point simultaneously maximized SO_2_ release and minimized H_2_O evaporation. To illustrate the range of trade-offs, three representative Pareto points were identified. The water-saving representative was defined as the Pareto point with the minimum *r*H_2_O, whereas the SO_2_-priority representative was defined as the point with the maximum *r*SO_2_. A balanced representative was selected as the Pareto point with the minimum Euclidean distance to the ideal point (1,0) after min–max normalization of the two objectives. These points are used only to illustrate the Pareto trade-off and are distinct from the final engineering recommendation obtained from the weighted screening analysis.

Combining the Pareto front with the normalized score produced a priority window of 26.25–30.25 kPa and 339.63–341.63 K (Figure 8b). The balanced optimization identified an engineering candidate near 28 kPa and 341 K. Considering the sparse experimental coverage and the absence of uncertainty propagation through the optimization, the result is reported as an exploratory operating neighborhood of approximately 26–30 kPa and 340–342 K rather than as a precisely defined operating window. This region is intended to guide confirmatory experiments and should not be interpreted as a confidence region or experimentally validated operating tolerance. The surface is continuous in this region, which remains close to the supporting observations. The window retains useful SO_2_ release while moderating evaporation and was carried forward to the dynamic calculation. It is not a universal set point: its location depends on rich-solution composition, objective weights, and surrogate accuracy.

#### 2.2.5. Dynamic Behavior and Material-Balance Consistency

An illustrative 120 min dynamic simulation was performed at *P* = 20 kPa and *T* = 340 K using *V*_0_ = 1 L, *c*Al = 15 g·L^−1^, *B* = 25%, and *c*SO_3_^2−^ = 0.06 mol·L^−1^. The calculation used a time step of 10 s and zero initial cumulative SO_2_ and H_2_O releases. The simulation coupled surrogate-rate predictions to recursive material balances so that sulfite inventory, liquid volume, and the apparent concentrations of nonvolatile species were updated as regeneration proceeded.

**Figure 9:**
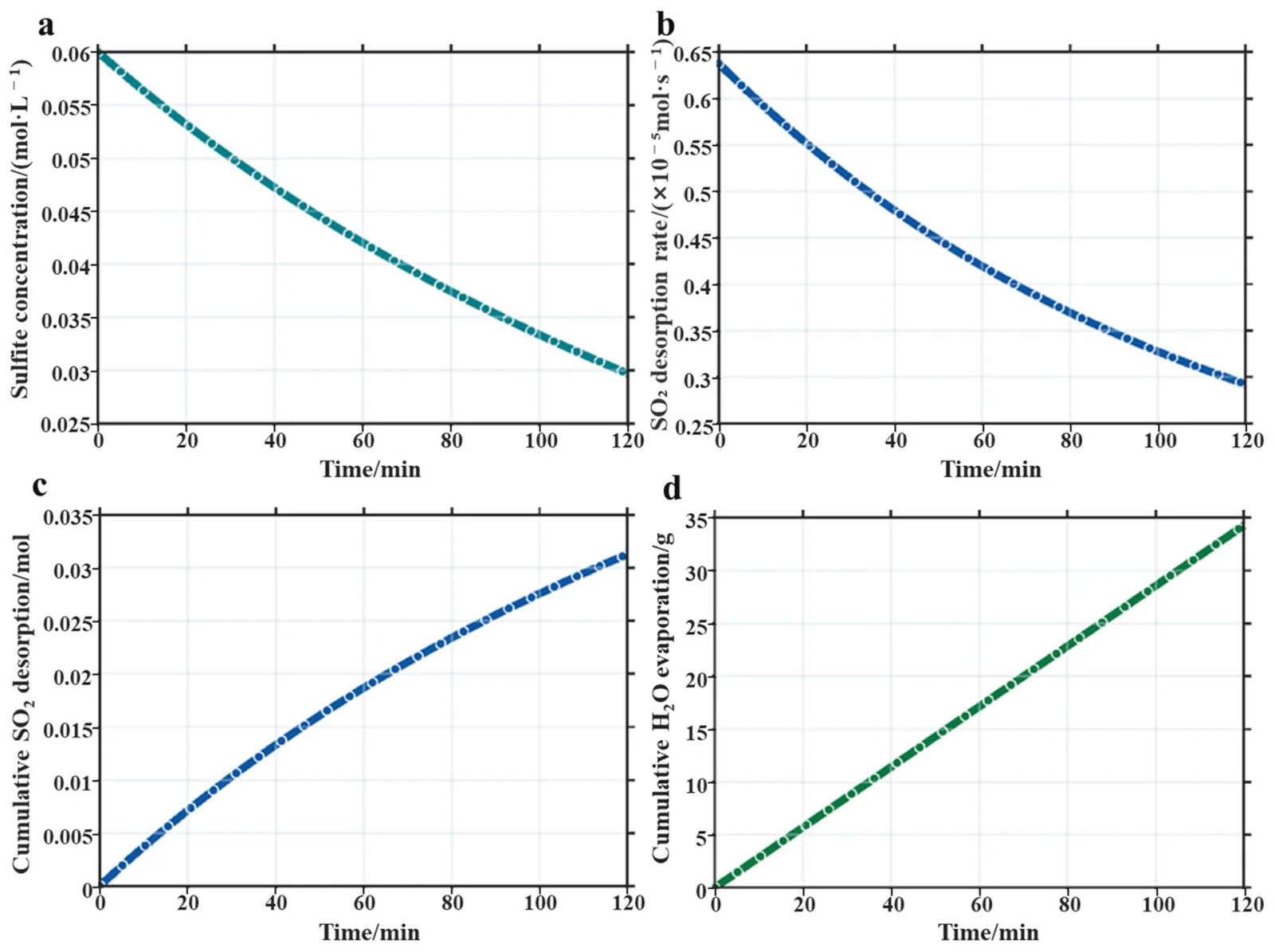
Changes during the 120 min dynamic simulation: (**a**) sulfite concentration, (**b**) instantaneous SO_2_ desorption rate, (**c**) cumulative SO_2_ desorption and (**d**) cumulative H_2_O evaporation.

As shown in Figure 9, *c*SO_3_^2−^ and the instantaneous SO_2_ rate declined throughout the simulation. A larger initial inventory supported rapid early release, but subsequent depletion progressively reduced the marginal SO_2_ recovered. Cumulative SO_2_ continued to increase at a diminishing rate. Cumulative H_2_O evaporation, by contrast, kept rising throughout the 120 min period. The divergence has a direct operating implication: extending a run can yield little additional SO_2_ while continuing to remove water and consume latent heat.

**Figure 10:**
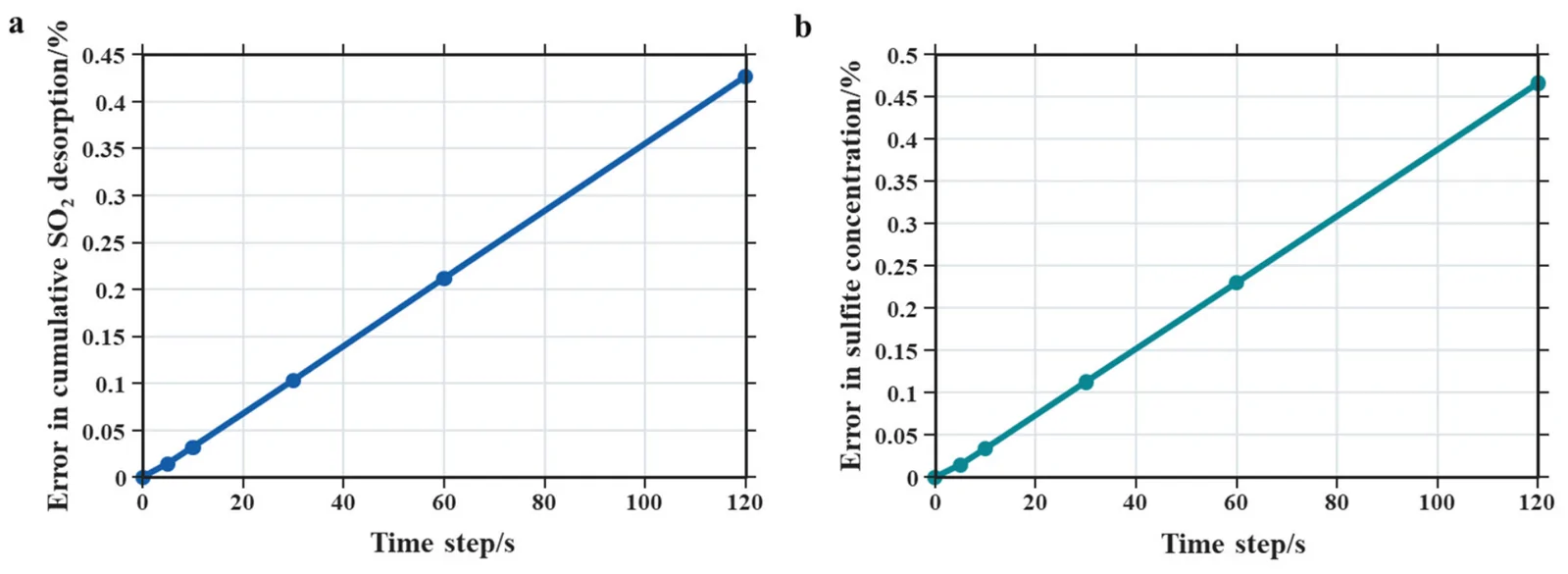
Material-balance error assessment for the dynamic simulation: (**a**) error in cumulative SO_2_ desorption and (**b**) error in sulfite concentration at different time steps.

Figure 10 shows low errors in cumulative SO_2_ release and sulfite-concentration updating at the tested time steps, with no pronounced accumulated drift. The recursive calculation was therefore numerically consistent with its material balances. This check establishes conservation within the algorithm, not external accuracy of the simulated trajectory, which still depends on the surrogate models and source data.

### 2.3. Limitations, Engineering Implications, and Future Research

The findings must be read within four boundaries. The finite, predominantly single-factor datasets provide uneven coverage and mainly support interpolation. Composition predictors were unavailable for the H_2_O response, so its two-variable form is empirical rather than mechanistic. The surrogates reproduce macroscopic rates without resolving equilibrium, mass-transfer, interfacial-renewal, or phase-change mechanisms. Finally, cross-validation and balance closure are internal checks; neither replaces an independent continuous-regeneration experiment. These qualifications are essential whenever small-data surrogates are used for process decisions [21,22,24].

Within those boundaries, the hierarchy improves on repeated one-factor screening by preserving the richer composition information for SO_2_, limiting the H_2_O model to observed predictors, and exposing the recovery–evaporation conflict. The identified window is therefore best used to prioritize confirmatory tests and preliminary design calculations. The dynamic result also shows that termination time is a control variable: once incremental SO_2_ recovery becomes small while evaporation persists, continued regeneration is unlikely to be attractive.

The present optimization does not include a complete process–energy balance. Therefore, H_2_O evaporation is interpreted as a solvent-loss response with qualitative energy implications rather than as a direct measure of total regeneration energy consumption.

Further work should use a structured design in which pressure, temperature, and composition vary jointly, with denser observations in the high-rate and high-sulfite regions. Expanding the H_2_O dataset across *c*Al, *B*, and *c*SO_3_^2−^ would test whether solution composition materially changes evaporation. Continuous or semicontinuous experiments should then verify the recommended window and time trajectories while measuring water loss, heat duty, and the absorption performance of the regenerated liquid. Hybrid models that impose equilibrium and mass-transfer constraints could improve interpretability, and propagation of surrogate uncertainty through the Pareto and dynamic calculations would allow future operating recommendations to be reported with confidence bounds.

## 3. Materials and Methods

### 3.1. Research Strategy

#### 3.1.1. Overall Research Design

The research strategy linked five tasks: response-specific problem definition, data preparation, surrogate-model training and validation, multiobjective operating analysis, and recursive dynamic simulation. Separate models were retained because the SO_2_ and H_2_O experiments did not contain the same predictors. This hierarchy uses all supported information for each response while avoiding artificial dependencies in the smaller H_2_O dataset, an approach consistent with data-driven process optimization under limited observations [21].

#### 3.1.2. Process Representation and Modeling Rationale

Vacuum regeneration reflects chemical-equilibrium shifts, gas–liquid transfer, and water phase change. In the absorber, dissolved SO_2_ hydrates and dissociates, while S(IV) is distributed among HSO_3_^−^, SO_3_^2−^, and sulfur-containing aluminum species. Lowering pressure or raising temperature favors molecular SO_2_ and its transfer to the gas phase. The principal acid–base relations are denoted by (1) and (2).

(1) $${\mathrm{SO}}_{2}{(g)+H}_{2}O(l)\;⇌H^{+}{+\mathrm{HSO}}_{3}^{-}$$

(2) $${\mathrm{HSO}}_{3}^{-}⇌H^{+}{+\mathrm{SO}}_{3}^{2-}$$

Water evaporates at the same time, as represented by (3). Lower pressure increases the gas-phase driving force, and higher temperature increases volatility; both changes can therefore raise the instantaneous SO_2_ and H_2_O rates.

H_2_O(l) ⇌ H_2_O(g) (3)

Absolute pressure affects phase equilibrium and gas composition but is not itself the SO_2_ mass-transfer driving force. The latter depends on the departure from gas–liquid equilibrium and the corresponding SO_2_ partial-pressure or concentration difference. The surrogate models do not resolve these quantities separately. Vapor generation may enhance interfacial renewal in the source stirred, heated apparatus, but this configuration-specific mechanism is not independently identified by the surrogate models. Evaporation can renew the gas–liquid interface and assist SO_2_ removal, but it also reduces liquid volume and concentrates nonvolatile species. Because the available single-factor data cannot identify all equilibrium, kinetic, and transport parameters, a full mechanistic model would be underconstrained. The surrogate hierarchy was selected to retain the observed response patterns while keeping model complexity proportional to the evidence.

### 3.2. Data Collection and Variable Definition

#### 3.2.1. Data Source and Response-Specific Datasets

No new experiments were performed in this study. All observations used for surrogate-model development were extracted from Chen’s study [16]; the related BAS-regeneration studies cited in Refs. [17,18,19] were used only for contextual discussion and contributed no observations to the present datasets. The source archive contains 37 plotted SO_2_ records: 8 from the pressure series, 9 from the temperature series, 6 from the aluminum-concentration series, 7 from the basicity series and 7 from the sulfite-concentration series. One identical predictor combination occurs in both the aluminum and basicity series and was consolidated before model evaluation, leaving 36 unique SO_2_ modeling inputs. The H_2_O dataset contains 17 distinct observations, comprising 8 pressure-series and 9 temperature-series conditions.

The source experiments used 250 mL of solution in a 500 mL three-neck flask, with 2% (*v*/*v*) ethylene glycol and stirring at 120 rpm for the analyzed series. Although the source reports at least three experimental repetitions per condition, individual replicate measurements are not available in the modeling archive. The sample sizes reported here therefore refer to the plotted experimental conditions used for model construction and validation, rather than to the total number of underlying replicate runs.

For the SO_2_ dataset, the sampled ranges were *P* = 14.26–45.34 kPa, *T* = 322.10–345.08 K, *c*Al = 15–65 g·L^−1^, *B* = 5–35%, and *c*SO_3_^2−^ = 0.02–0.60 mol·L^−1^. For the H_2_O dataset, the sampled ranges were *P* = 14.31–45.29 kPa and *T* = 322.10–345.08 K. The complete sampled levels, frequencies and descriptive statistics for each predictor are reported in Appendix A.2. Because the source experiments were predominantly single-factor designs, these marginal ranges should not be interpreted as uniform coverage of the corresponding multidimensional input space.

For SO_2_ desorption, absolute pressure (*P*), temperature (*T*), aluminum concentration (*c*Al), basicity (*B*), and sulfite concentration (*c*SO_3_^2−^) were predictors, and the SO_2_ desorption rate (*r*SO_2_) was the response. (4) denotes this mapping.

(4) $$r_{{\mathrm{SO}}_{2}}=f(P,T,c_{\mathrm{Al}},B,c_{{\mathrm{SO}}_{3}^{2-}})$$

The evaporation measurements supported a narrower relationship: *P* and *T* were used to predict the H_2_O evaporation rate (*r*H_2_O), as denoted by (5). Composition variables were not added because no corresponding H_2_O observations were available to verify their effects.

(5) $$r_{H_{2}O}=g(P,T)$$

#### 3.2.2. Variables and Physical Interpretation

The variables used in this paper and their physical explanations are listed in Table 1.

### 3.3. Data Analysis Methods

#### 3.3.1. Data Preprocessing

Before model fitting, the extracted datasets were checked for missing values, duplicated predictor combinations and unit consistency. One duplicated SO_2_ predictor combination was consolidated into a single modeling entry while retaining its source identity. The stored numerical responses were converted to physical molar rates according to *r*SO_2_ = 10^−5^ *y*SO_2_ and *r*H_2_O = 10^−4^ *y*H_2_O. Predictor standardization was applied where required by the corresponding surrogate model, using statistics estimated from the relevant training data. Each model used only the variables in its response-specific mapping. No additional variables were imputed for observations for which corresponding source measurements were unavailable. Predictors were standardized by their sample means and standard deviations according to (6),

(6) $$x_{j}^{\prime }=\frac{x_{j}-\mu_{j}}{\sigma_{j}}$$

where *x_j_*, *μ_j_*, and *σ_j_* are the original value, mean, and standard deviation of predictor *j*. Scaling places variables with different units on comparable numerical ranges and improves the stability of distance- and kernel-based methods, particularly SVR and GPR.

#### 3.3.2. Candidate Surrogate Models and Performance Criteria

The candidate set comprised multiple linear regression, polynomial regression, support vector regression (SVR), a regression tree, an ensemble of regression trees, and GPR. The linear and polynomial forms served as interpretable benchmarks. SVR and tree-based methods accommodated nonlinear structure [25,26], while GPR was included for its flexibility with smooth, small-sample process data [22,23]. Together, these models cover parametric, kernel, partition, ensemble, and probabilistic regression families used in process data science [24].

For GPR, an ARD Matérn-5/2 kernel was selected for the SO_2_ response and an ARD squared-exponential kernel for the H_2_O response. Both GPR models used a constant basis function, standardized predictors, and exact fitting and prediction. Complete implementation settings and kernel definitions are provided in Appendix A.1.

Prediction quality was evaluated with root-mean-square error (RMSE), mean absolute error (MAE), and the coefficient of determination (R^2^), defined by (7)–(9).

(7) $$\mathrm{RMSE}=\sqrt{\frac{1}{n}\sum_{i=1}^{n}{\left(y_{i}-{\hat{y}}_{i}\right)}^{2}}$$

(8) $$\mathrm{MAE}=\frac{1}{n}\sum_{i=1}^{n}\left|y_{i}-{\hat{y}}_{i}\right|$$

(9) $$R^{2}=1-\frac{\sum_{i=1}^{n}{\left(y_{i}-{\hat{y}}_{i}\right)}^{2}}{\sum_{i=1}^{n}{\left(y_{i}-\bar{y}\right)}^{2}}$$

where *y_i_* and ${\hat{y}}_{i}$ are measured and predicted values, $\bar{y}$ is the observed mean, and *n* is the number of samples. RMSE emphasizes large residuals, MAE reports average absolute deviation, and R^2^ indicates how much observed variation the model reproduces.

No model was accepted on a single metric. Error statistics were considered together with surface continuity and the physical direction of the pressure and temperature responses. This combined screen was intended to reject models that fit the available points but behaved implausibly between them.

#### 3.3.3. Cross-Validation and Physical-Consistency Assessment

Validation strategy was selected according to the available sample size for each response. Five-fold cross-validation was applied to the SO_2_ dataset comprising 36 unique modeling observations, whereas leave-one-out cross-validation (LOOCV) was used for the smaller H_2_O dataset comprising 17 observations, thereby retaining the maximum possible number of samples for model fitting in each iteration. For each candidate model, predictive performance was evaluated from held-out observations using RMSE, MAE and R^2^, together with inspection of the cross-validated residuals and the physical consistency of the predicted responses to pressure and temperature. Model selection therefore considered both predictive accuracy and physically plausible response behavior within the sampled domain.

For the selected GPR models, predictive uncertainty in the observed-versus-predicted plots was quantified using pointwise 95% response prediction intervals. To maintain consistency with LOOCV, the interval for each observation was calculated from the same leave-one-out model used to obtain its held-out prediction. For each held-out input *x_i_*, the fitted GPR model provided a predictive mean *ŷ_i_*, predictive standard deviation *σ_pred,i_*, and a two-sided response prediction interval at α = 0.05. The resulting intervals include the fitted observation-noise component and are conditional on the fitted model and hyperparameters. They therefore quantify model-conditioned predictive uncertainty for an individual response rather than uncertainty in the predicted mean. For continuous response-curve visualization, including Figure 5c, pointwise 95% GPR response prediction intervals were calculated at each query condition using the GPR model fitted to the available SO_2_ dataset, with the same α = 0.05 definition.

Permutation feature importance was evaluated using the fitted response models. After negative predictions were clipped to zero, the baseline training-set mean squared error (MSE) was calculated. Each predictor was then independently permuted 40 times without refitting the model. Its importance was defined as the nonnegative mean increase in MSE relative to the baseline and normalized by the sum of the importance values across predictors within the same response model. A fixed random seed of 42 was used for reproducibility. Because the resulting values are model-dependent, in-sample diagnostics, they were interpreted as relative predictive importance within each response model rather than as directly comparable effect sizes across responses.

#### 3.3.4. Multiobjective Operating-Condition Analysis

Pressure and temperature act on both objectives in the same direction: lower *P* and higher *T* promote SO_2_ release but also increase water evaporation. Maximizing *r*SO_2_ alone would therefore favor conditions with a potentially excessive solvent and energy penalty. This concern is material because water vaporization has been reported to account for more than 85% of BAS regeneration energy use [16]. The validated surrogates were consequently combined in the two-objective problem represented by (10), which maximizes *r*SO_2_ while minimizing *r*H_2_O.

(10) $$\max\;r_{{\mathrm{SO}}_{2}},\min\;r_{H_{2}O}$$

Operating conditions were screened using a weighted multi-component objective incorporating SO_2_ recovery, selectivity, H_2_O evaporation, and model support. For the balanced scenario, the corresponding weighting coefficients were *w*SO_2_ = 0.42, *w*Sel = 0.18, *w*H_2_O = 0.25, and *w*Sup = 0.15. These weights represent the relative priorities assigned to the four optimization components and were used as process-screening parameters rather than plant-specific economic coefficients.

The search was performed on a uniform 41 × 41 pressure–temperature grid covering *P* = 14.31–45.29 kPa and *T* = 322.10–345.08 K, corresponding to 1681 candidate points and grid spacings of approximately 0.7745 kPa and 0.5745 K, respectively. The composition was fixed at *c*Al = 15.00 g·L^−1^, *B* = 25.00%, and *c*SO_3_^2−^ = 0.0600 mol·L^−1^.

Candidate points were further screened using an H_2_O-evaporation constraint based on the 75th percentile of the predicted H_2_O rates and a model-support criterion. Sensitivity to the weighting preference was examined using SO_2_-priority, balanced, and water-saving-priority scenarios. The complete optimization settings are provided in Appendix A.3.

Water evaporation has qualitative energy implications, but the present optimization does not calculate regeneration heat duty, vacuum-system work, condenser duty, sensible heat, or energy recovery. The resulting operating condition therefore represents an SO_2_ recovery–water-loss compromise rather than an energy optimum.

#### 3.3.5. Dynamic Simulation and Material-Balance Updating

The source response targets are average total molar rates for the experimental apparatus. In the recursive illustration, their predictions at the current composition were treated as effective instantaneous rates under an explicit quasi-steady approximation. The surrogate models were fitted and evaluated using scaled numerical responses, denoted by *y*SO_2_ and *y*H_2_O. Here, *y*SO_2_ represents the SO_2_ desorption rate in units of 10^−5^ mol·s^−1^, whereas *y*H_2_O represents the H_2_O evaporation rate in units of 10^−4^ mol·s^−1^. The corresponding physical rates used in the material balances were therefore calculated as *r*SO_2_ = 10^−5^ *y*SO_2_ and *r*H_2_O = 10^−4^ *y*H_2_O. Their products with Δ*t* updated cumulative SO_2_ release and water loss, as represented by (11) and (12). For each time step, Δ*n*SO_2_ and Δ*n*H_2_O denote the molar amounts released during that interval, whereas *N*SO_2_ and *N*H_2_O denote the corresponding cumulative molar releases from the start of the simulation. All *n* and *N* terms are expressed in mol. Sulfite inventory and apparent concentration were recalculated before the next prediction, allowing the rate to respond to depletion.

(11) $$\Delta n_{{\mathrm{SO}}_{2}}=\min[r_{{\mathrm{SO}}_{2}}(t)\Delta t,n_{S}(t)]\;N_{{\mathrm{SO}}_{2}}(t+\Delta t)=N_{{\mathrm{SO}}_{2}}(t)+\Delta n_{{\mathrm{SO}}_{2}}$$

(12) $$\Delta n_{H_{2}O}=r_{H_{2}O}(t)\Delta t\;N_{H_{2}O}(t+\Delta t)=N_{H_{2}O}(t)+\Delta n_{H_{2}O}$$

Water moles were converted to mass using Δ*m*H_2_O = MH_2_OΔ*n*H_2_O, with MH_2_O = 18.01528 g·mol^−1^. (14) then updates volume using ρH_2_O = 1000 g·L^−1^, so V is in litres. Sulfur inventory follows *n*S(*t* + Δ*t*) = *n*S(*t*) − Δ*n*SO_2_, and the next concentrations are *c*SO_3_ = *n*S/V and *c*Al = *m*Al, 0/V. Aluminum mass and basicity remain constant. This recursion links instantaneous predictions to the evolving liquid state instead of assuming a constant composition throughout regeneration.

(13) $$V(t+\Delta t)=V(t)-\frac{M_{H_{2}O}\Delta n_{H_{2}O}}{\rho_{H_{2}O}}$$

The source experiments used a 0.25 L liquid inventory, whereas the illustrative dynamic calculation used *V*_0_ = 1 L. Applying the source-apparatus total-rate surrogate to this larger inventory is therefore an explicit modeling assumption rather than a validated volume-scaling relation. Likewise, treating predicted average rates as effective instantaneous rates does not constitute an experimentally established average-to-instantaneous conversion.

## 4. Conclusions

This study asked how simultaneous SO_2_ desorption and H_2_O evaporation can be predicted and balanced during vacuum regeneration of SO_2_-rich BAS solution when the available datasets differ in size and dimensionality. Treating the outputs as related but distinct response problems resolved that mismatch without discarding supported SO_2_ composition variables or inventing composition effects for H_2_O. The resulting hierarchy connects instantaneous-rate prediction, operating trade-off analysis, and time-dependent material balances in one process-screening framework.

GPR-M52-ARD was the most accurate SO_2_ surrogate (RMSE = 0.0631; R^2^ = 0.9746), while GPR-ARDSE best represented H_2_O evaporation (RMSE = 0.0793; R^2^ = 0.9928). The SO_2_ model was governed chiefly by sulfite concentration (61.7%), temperature (25.3%), and pressure (11.6%); the H_2_O model attributed 53.7% of predictive importance to pressure and 46.3% to temperature. These rankings distinguish the changing sulfur inventory from the two operating variables shared by both responses.

The principal process finding is that stronger vacuum and higher temperature intensify recovery and water loss together. The largest pressure-driven gains occurred as pressure fell from 45 kPa to about 15 kPa, after which deeper vacuum produced diminishing benefits. Pareto analysis translated this conflict into a model-supported compromise of 26–30 kPa and 340–342 K. The 120 min simulation added a time dimension: SO_2_ release slowed as sulfite was depleted, but water loss continued. Efficient operation should therefore coordinate pressure, temperature, and stopping time instead of maximizing the instantaneous SO_2_ rate.

The study contributes a transparent method for drawing operating guidance from limited, uneven process data. For BAS desulfurization, it shifts regeneration assessment from a single recovery metric toward the marginal value of additional SO_2_ recovery relative to continuing water loss and its associated energy implications. More broadly, the response-specific strategy may be useful when a desired recovered product and an energy-intensive solvent-vaporization response are measured with different predictor sets.

The conclusions apply only within the pressure, temperature, and composition ranges represented by the source experiments. The optimization held rich-solution composition fixed, and the dynamic trajectory was used for trend analysis rather than as an independently validated industrial prediction. The finite, mostly single-factor datasets remain uneven; composition effects on H_2_O evaporation are unmeasured; mechanistic equilibrium and transport constraints are absent; and model uncertainty was not propagated through optimization and simulation. Because total regeneration energy was not calculated, the identified operating region should not be interpreted as an energy optimum. Future research should therefore collect jointly varied data, validate the window in continuous or semicontinuous equipment, measure heat duty and regenerated-liquid performance, and develop uncertainty-aware hybrid models. These steps would test transferability while preserving the central conclusion that BAS regeneration is a coupled SO_2_-recovery/water-loss problem.

## Figures and Tables

**Table 1:** Model variables, units and physical interpretation.

Symbol	Variable	Unit	Physical Interpretation
*P*	Absolute pressure	kPa	Affects phase equilibrium and gas composition
*T*	Temperature	K	Affects volatility and liquid-phase equilibrium
*c*Al	Aluminum concentration	g·L^−1^	Represents the concentration of aluminum-buffer species
*B*	Basicity	%	Influences acidity/alkalinity and the distribution of S(IV) species
*c*SO_3_^2−^	Sulfite concentration	mol·L^−1^	Represents the releasable sulfur content
*r*SO_2_	SO_2_ desorption rate	mol·s^−1^	Average total molar rate used as the SO_2_ surrogate response
*r*H_2_O	H_2_O evaporation rate	mol·s^−1^	Average total molar rate used as the H_2_O surrogate response

## Data Availability

Data are contained within the article. The analysis used vacuum-regeneration data reported in the cited source studies. The numerical data and reproducibility information underlying the surrogate-model analyses are provided in Appendix A, including the model inputs and responses, physical units, response scaling factors, source mappings, and data-preprocessing and digitization procedures.

## Appendix A

### Appendix A.1

All surrogate-model calculations were implemented in MATLAB R2021a 9.10.0.1602886 using the Statistics and Machine Learning Toolbox 12.1. The candidate-model set comprised multiple linear regression, second-order polynomial regression, support vector regression (SVR), a standalone regression tree, an LSBoost regression-tree ensemble, and Gaussian process regression (GPR).

The polynomial regression model was specified as a full second-order model containing linear terms, squared terms, and pairwise interaction terms. SVR employed a Gaussian radial-basis-function (RBF) kernel. Predictors were standardized before model fitting, and the kernel scale was determined automatically rather than fixed at a user-specified numerical value.

The standalone regression tree was fitted using fitrtree, with MinLeafSize = 4, MaxNumSplits = ntrain − 1, and surrogate splitting disabled (Surrogate = “off”), where *n*_train_ denotes the number of observations available in the corresponding training set. The LSBoost ensemble comprised 200 regression trees, with a maximum of five splits per tree and a learning rate of 0.05.

The selected SO_2_ surrogate employed an automatic-relevance-determination (ARD) Matérn-5/2 covariance function, whereas the selected H_2_O surrogate employed an ARD squared-exponential covariance function. Both GPR models used a constant basis function, standardized predictors, and exact fitting and prediction. GPR hyperparameters were estimated internally using MATLAB fitrgp; no user-defined hyperparameter bounds were imposed.

For standardized predictor vectors *z* and *z*’, the ARD distance was defined as

$$d^{2}(z,z^{\prime })={\sum_{j=1}^{p}\left(\frac{z_{j}-{z_{j}}^{\prime }}{l_{j}}\right)}^{2},$$

where $l_{j}$ is the characteristic length scale associated with predictor *j*. The ARD squared-exponential covariance function was expressed as

$$k_{\mathrm{SE}}(z,z^{\prime })=\sigma_{f}^{2}\exp(-\frac{d^{2}}{2}),$$

whereas the ARD Matérn-5/2 covariance function was

$$k_{M52}(z,z^{\prime })=\sigma_{f}^{2}(1+\sqrt{5}d+\frac{5d^{2}}{3})\exp(-\sqrt{5}d),$$

where $\sigma_{f}^{2}$ denotes the signal variance. The ARD formulation assigns a separate characteristic length scale to each predictor.

Model predictive performance was assessed using leave-one-out cross-validation. Random seeds were required only for stochastic post-processing procedures and were not used to define the deterministic LOOCV partition. A random seed of 42 was used for the permutation feature-importance analysis, and a seed of 20,260,426 was used for the bootstrap uncertainty analysis. Fixing these seeds ensures that the same permutation and resampling sequences can be reproduced in repeated calculations.

### Appendix A.2

This appendix summarizes the empirical distributions of the predictors used for model development. Statistics are calculated from the 36 unique SO_2_ input combinations and 17 H_2_O observations after consolidation of the repeated SO_2_ predictor combination. For each predictor, we report the sampled range, mean ± sample standard deviation, and the frequency of every observed level. These statistics describe the distribution and concentration of the model inputs and do not represent replicate-level measurement uncertainty. Because the underlying experiments were predominantly single-factor studies, marginal predictor ranges do not imply uniform or complete joint coverage of the multidimensional input space.

*SO_2_ input distribution*

Pressure (kPa): range 14.26–45.34; mean ± SD 21.427 ± 5.2953. Levels (count): 14.26 (1); 17.27 (1); 20 (28); 20.23 (1); 23.33 (1); 26.34 (1); 29.3 (1); 35.31 (1); 45.34 (1).

Temperature (K): range 322.1–345.08; mean ± SD 338.47 ± 4.6307. Levels (count): 322.1 (1); 325.14 (1); 328.13 (1); 331.13 (1); 334.08 (1); 337.12 (1); 340 (27); 340.11 (1); 342.13 (1); 345.08 (1).

Aluminum (g·L^−1^): range 15–65; mean ± SD 19.167 ± 11.802. Levels (count): 15 (31); 25 (1); 35 (1); 45 (1); 55 (1); 65 (1).

Basicity (%): range 5–35; mean ± SD 24.028 ± 4.9018. Levels (count): 5 (1); 10 (1); 15 (1); 20 (1); 25 (30); 30 (1); 35 (1).

Sulfite (mol·L^−1^): range 0.02–0.6; mean ± SD 0.24389 ± 0.25612. Levels (count): 0.02 (1); 0.04 (1); 0.06 (18); 0.08 (1); 0.1 (1); 0.12 (1); 0.14 (1); 0.6 (12).

*H_2_O input distribution*

Pressure (kPa): range 14.31–45.29; mean ± SD 23.025 ± 7.4787. Levels (count): 14.31 (1); 17.27 (1); 20 (9); 20.38 (1); 23.23 (1); 26.34 (1); 29.35 (1); 35.26 (1); 45.29 (1).

Temperature (K): range 322.1–345.08; mean ± SD 336.77 ± 6.4065. Levels (count): 322.1 (1); 325.14 (1); 328.13 (1); 331.13 (1); 334.08 (1); 337.12 (1); 340 (8); 340.11 (1); 342.13 (1); 345.08 (1).

*Data provenance, transcription and preprocessing*

This part records the model input and response variables, physical units, response scaling factors and corresponding source information used for surrogate-model construction. Numerical observations were digitized from the source figures in Ref. [16]. For each figure, the horizontal and vertical axes were calibrated against the reported tick values, after which the experimental data markers were manually identified and extracted. Exported values were retained at a precision consistent with the resolution of the original figures.

The extracted records were checked for missing entries, duplicated predictor combinations and unit consistency. One duplicated SO_2_ predictor combination was consolidated into a single modeling entry while preserving its original source mapping. Scaled numerical responses were converted to physical molar rates using *r*SO_2_ = 10^−5^ *y*SO_2_ and *r*H_2_O = 10^−4^ *y*H_2_O. Predictor standardization was applied only where required by the corresponding surrogate model.

### Appendix A.3

The operating-condition search was performed over a uniform two-dimensional pressure–temperature grid. Pressure ranged from 14.31 to 45.29 kPa and temperature from 322.10 to 345.08 K. Forty-one equally spaced levels were used for each variable, producing a 41 × 41 grid containing 1681 candidate operating points. The resulting pressure and temperature increments were approximately 0.7745 kPa and 0.5745 K, respectively.

For all candidate evaluations, the solution composition was fixed at an aluminum concentration of 15.00 g·L^−1^, a basicity of 25.00%, and a sulfite concentration of 0.0600 mol·L^−1^. Thus, the operating-condition analysis examined pressure–temperature effects at a common baseline composition rather than simultaneously optimizing solution composition.

The balanced optimization objective contained four weighted components representing SO_2_ recovery, selectivity, H_2_O evaporation, and model support. Their respective weighting coefficients were

$$w_{{\mathrm{SO}}_{2}}=0.42\;w_{\mathrm{Sel}}=0.18\;w_{H_{2}O}=0.25\;w_{\mathrm{Sup}}=0.15$$

with the four coefficients summing to unity. These coefficients represent relative screening priorities and should not be interpreted as plant-specific economic weights.

Candidate operating points were subjected to additional feasibility screening. The H_2_O-evaporation constraint was defined relative to the 75th percentile of the predicted H_2_O evaporation rates across the candidate population. A model-support criterion was additionally applied to reduce selection of conditions judged to have insufficient support from the available modeling data.

Under the balanced weighting scheme, the engineering optimum occurred at approximately *P* = 28.25 kPa and *T* = 341.63 K. The corresponding recommended operating window was *P* = 26.25–30.25 kPa and *T* = 339.63–341.63 K.

Sensitivity to the weighting preference was examined using three optimization scenarios: SO_2_-priority, balanced, and water-saving-priority. These scenarios were used to evaluate the extent to which the selected operating conditions depended on the relative priorities assigned to product recovery and water conservation. The balanced scenario was used as the reference case for the operating window reported in the main text.
